# Molecular Analysis of Bacterial Communities and Detection of Potential Pathogens in a Recirculating Aquaculture System for *Scophthalmus maximus* and *Solea senegalensis*

**DOI:** 10.1371/journal.pone.0080847

**Published:** 2013-11-21

**Authors:** Patrícia Martins, Daniel F. R. Cleary, Ana C. C. Pires, Ana Maria Rodrigues, Victor Quintino, Ricardo Calado, Newton C. M. Gomes

**Affiliations:** Department of Biology and CESAM, University of Aveiro, Aveiro, Portugal; National Science and Technology Development Agency, Thailand

## Abstract

The present study combined a DGGE and barcoded 16S rRNA pyrosequencing approach to assess bacterial composition in the water of a recirculating aquaculture system (RAS) with a shallow raceway system (SRS) for turbot (*Scophthalmus maximus*) and sole (*Solea senegalensis*). Barcoded pyrosequencing results were also used to determine the potential pathogen load in the RAS studied. Samples were collected from the water supply pipeline (Sup), fish production tanks (Pro), sedimentation filter (Sed), biofilter tank (Bio), and protein skimmer (Ozo; also used as an ozone reaction chamber) of twin RAS operating in parallel (one for each fish species). Our results revealed pronounced differences in bacterial community composition between turbot and sole RAS, suggesting that in the systems studied there is a strong species-specific effect on water bacterial communities. *Proteobacteria* was the most abundant phylum in the water supply and all RAS compartments. Other important taxonomic groups included the phylum *Bacteriodetes*. The saltwater supplied displayed a markedly lower richness and appeared to have very little influence on bacterial composition. The following potentially pathogenic species were detected: *Photobacterium damselae* in turbot (all compartments), *Tenacibaculum discolor* in turbot and sole (all compartments), *Tenacibaculum soleae* in turbot (all compartments) and sole (Pro, Sed and Bio), and *Serratia marcescens* in turbot (Sup, Sed, Bio and Ozo) and sole (only Sed) RAS. Despite the presence of these pathogens, no symptomatic fish were observed. Although we were able to identify potential pathogens, this approach should be employed with caution when monitoring aquaculture systems, as the required phylogenetic resolution for reliable identification of pathogens may not always be possible to achieve when employing 16S rRNA gene fragments.

## Introduction

The growing worldwide demand for fish has prompted research towards intensive aquaculture. Innovative technologies, such as recirculating aquaculture systems (RAS) and shallow raceway systems (SRS) [1], have been developed to improve the efficiency and sustainability of intensive aquaculture practices. RAS basically consists of the recycling and re-using of production water thus reducing adverse environmental impacts associated with water usage and release of nutrient-rich effluents [2-4]. SRS represent an improvement of common raceways, as they have an optimized hydrodynamic performance due to their low water level and plug-flow pattern thus enabling producers to employ higher fish stocking densities [1]. The organic matter resulting from unconsumed food and fish metabolites is recycled in RAS by a diverse array of microbes. Nitrogen-containing organic molecules are decomposed into ammonia by heterotrophic bacteria, with ammonia subsequently being converted into nitrite and nitrite into nitrate by nitrifying bacteria in biological filters. Given the key role played by these microorganisms in the operation of RAS, it is no surprise that most studies addressing bacterial communities in these production systems have mainly focused on biological filters [5,6]. However, there is a lack of information on the overall diversity and composition of bacterial communities in the different components of these intensive aquaculture systems. An in-depth analysis of these microbial communities will provide quantitative and qualitative outputs that should allow us to obtain a comprehensive definition of the 'standard' microbiome of a RAS. In turn this information can improve our ability to understand and control the microbial quality of production systems and reduce the risks associated with disease outbreak.

Traditionally, conventional microbiological techniques, such as culture-based approaches, serology and histology, have been used for pathogen detection in aquaculture. However, these techniques are often laborious and time-consuming. The development of molecular tools has allowed researchers to overcome these limitations [e.g. polymerase chain reaction (PCR) and real-time PCR (RT-PCR)] [7,8]. However, the application of these technologies depends on the selection of a range of pathogens to be targeted by the assay and, therefore, new or unexpected pathogens will not be detected. Alternatively, molecular fingerprint analyses of microbial communities [e.g. PCR - denaturing gradient gel electrophoresis (PCR-DGGE)] enable us to profile complex microbial communities in a range of environments [9-11]. Overall, these community fingerprint techniques are cost-effective, allow a fast characterization of different microbial assemblages in multiple samples and can easily be used to monitor microbial community structure in fish farms [12]. However, although this approach can provide important information on the structural diversity of microbes at different taxonomic levels (group specific PCR-DGGE for different kingdom, phylum, class, order, family, genus and species) [13], no information concerning the identity of microbial populations is provided.

In order to overcome these constraints, it is now possible to employ high throughput sequencing technologies (e.g. pyrosequencing) to achieve an in depth compositional analysis of complex microbial communities with an unprecedented level of resolution [14-16]. Additionally, these technologies can be especially interesting for monitoring fish disease in aquaculture systems. However, high throughput sequencing technologies demand specialized personnel and high-performance computing resources, and thus may not be readily available for most fish producers. Alternatively, DGGE can be used as a rapid proxy for the determination of compositional variation among different samples and/or experimental treatments. In this way, researchers can have a rapid overall characterization of the microbial communities being studied through DGGE and, at the same time, select the best strategy for sequencing analysis [15,17]. Here, for the first time, a DGGE - barcoded pyrosequencing approach was applied to explore the diversity of bacterial communities and detect potential fish pathogens in an intensive aquaculture operating RAS (with a SRS) for the production of *Scophthalmus maximus* (turbot) and *Solea senegalensis* (sole). The results obtained are critically discussed to highlight the advantages and limitations of this approach for the detection and characterization of bacterial pathogen assemblages in RAS.

## Materials and Methods

### Study site

**Figure 1:**
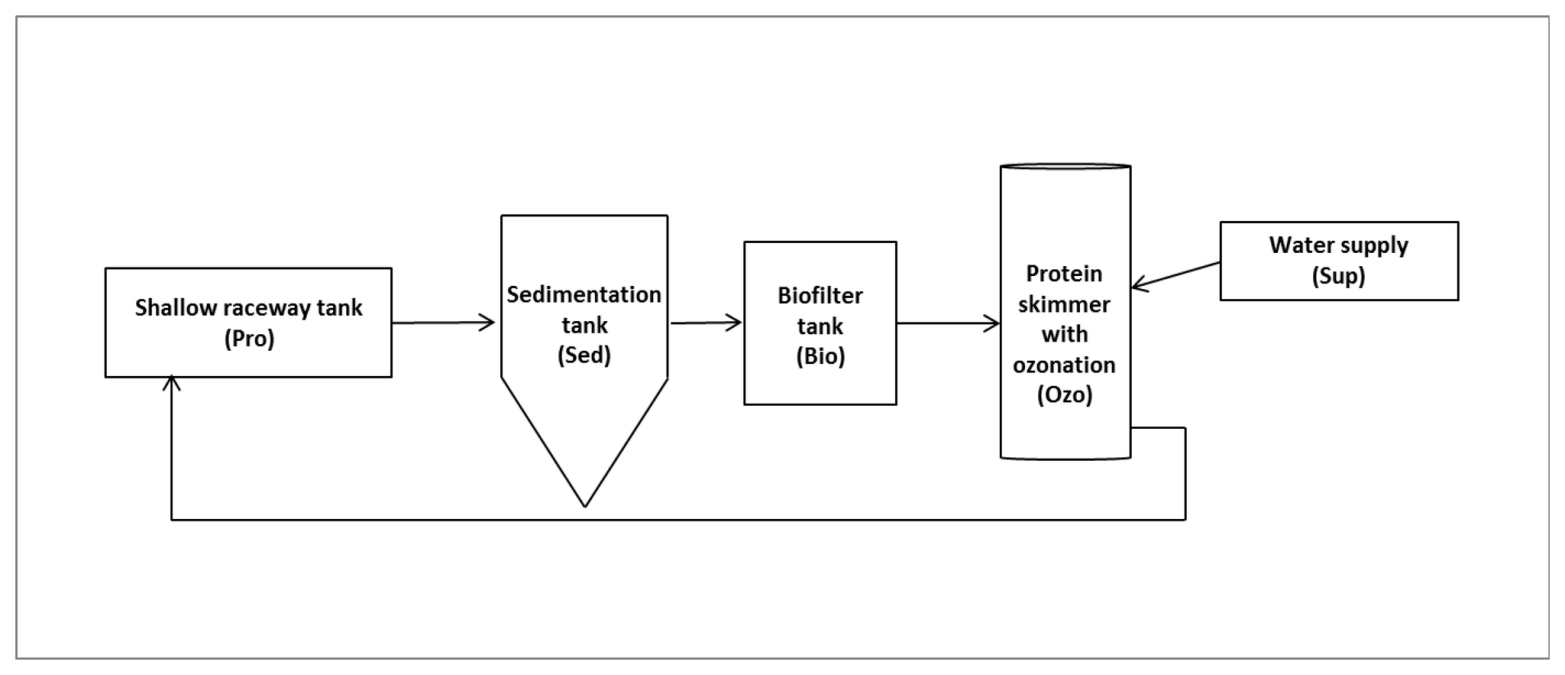
Schematic representation of the sampling compartments in RAS. Water circuit of the recirculating aquaculture system studied (not to scale).

The present study was carried out in a turbot (*S. maximus*) and sole (*S. senegalensis*) super-intensive fish farm located in northern Portugal, which employed RAS technology combined with SRS. Briefly, saltwater was pumped from a well and was strongly aerated and sand-filtered before entering each RAS; the water was recycled as it passed from the production tanks to the sedimentation tank where mechanical filtration was performed. The water then flowed to the biofilter tank for biological filtration and subsequently to the protein skimmer, which was also used as an ozone reaction chamber (Figure 1). 

### Sampling and DNA extraction

In RAS the external environmental parameters, such as temperature and natural photoperiod, have no influence on the system. Therefore, RAS display little to no seasonal variability in water parameters, allowing us to have a representative sample from the system by sampling at a single time point. Four sampling compartments for each species (*S. maximus* and *S. senegalensis*) were sampled: 1) shallow raceway tank (Pro) (containing fishes ~380 days old, weighing approximately 200 g to 300 g and densities with about 15 Kg/m^2^), 2) sedimentation tank (Sed), 3) biofilter tank (Bio) and 4) ozone tank (acting both as protein skimmer and ozone reaction chamber) (Ozo). We also sampled the water supply pipeline (Sup), which was the same for both RAS systems. The turbot and sole RAS were fully independent. 

Water samples (three replicates) were collected at different parts of the tank in each RAS compartment studied. Ammonia (NH_3_), nitrites (NO_2_^-^), nitrates (NO_3_^-^), bromine residuals (BR2), sulfates (SO_4_^2-^) and phosphates (PO_4_^3–^) were determined following the 8507, 8016 and 8155 methods described in the Hach Spectrophotometer (DR 2800) standard analytical procedures and according to EPA Method 300.1 and 351.2. Total organic carbon analysis (TOC) in the water was performed according to the European Norm 1484. Conventional physical-chemical parameters (temperature, pH, dissolved oxygen (DO), suspended particles and salinity) were also recorded.

For bacterial community analysis, water samples (250 ml) were ﬁltered through 0.2 µm pore polycarbonate membranes (Poretics, Livermore, CA, USA) and total community (TC) DNA extraction was performed directly on the filter using an E.Z.N.A Soil DNA Extraction kit (Omega Bio-Tek, USA) following the manufacturer's instructions.

### PCR-DGGE bacterial community fingerprinting

In this study DGGE fingerprinting was used as a rapid tool to determine compositional variation among bacterial communities in different samples prior to barcoded pyrosequencing [17]. After statistical analysis of DGGE profiles, we applied a more-in-depth compositional barcoded pyrosequencing analysis of composite samples. Amplified 16S rRNA gene fragments suitable for bacterial DGGE fingerprints of total microbial community DNA samples were obtained using a nested approach following Gomes et al. [18]. The V6-V8 regions of bacterial 16S rDNA fragments were amplified using the primers set 27F and 1494R [19,20] and 968GC - 1378R [21], for the first and second PCR, respectively. The PCRs were conducted in a Professional Thermocycler (Biometra). Positive and negative controls were run for each PCR.

DGGE was performed on a DCode Universal Mutation Detection System (Bio-Rad, Hercules, CA, USA). Samples were loaded onto 8% (w/v) polyacrylamide gels in 1x Tris-acetate-EDTA (TAE) with the denaturing gradient ranging from 40% to 58% (100% denaturant contains 7 M urea and 40% formamide) and performed at 58 °C at 160 V during 16 hours. Gels were silver-stained according to Byun et al. [22], except for the stop solution that in our case was replaced by a Na_2_CO_3_ solution. The image was acquired using the Epson perfection V700 Photo Scanner.

### Barcoded pyrosequencing

A barcoded pyrosequencing approach was used for in-depth compositional analyses of bacterial communities in turbot and sole RAS intensive cultures. Prior to pyrosequencing, TC-DNA of all three replicates of each sampling compartment was combined, forming one DNA library for each stage of the production system. This procedure was performed for the water supply and both fish species (*S. maximus* and *S. senegalensis*). The V3-V4 region of bacterial 16S rRNA gene amplicons were amplified using barcoded fusion primer V3 Forward (5´ -ACTCCTACGGGAGGCAG-3’) and V4 Reverse (5´ -TACNVRRGTHTCTAATYC-3’) with the Roche 454 titanium sequencing adapters. Pyrosequencing libraries were generated by the 454 Genome Sequencer FLX platform (Roche Diagnostics Ltd., West Sussex, United Kingdom) [23,24]. PicoGreen dsDNA quantitation kit (Invitrogen, Life Technologies, Carlsbad, California, USA) was used to quantify the PCR product, and was pooled at equimolar concentrations and sequenced in the A direction with GS 454 FLX Titanium chemistry, according to the manufacturer’s instructions (Roche, 454 Life Sciences, Brandford, CT, USA).

### Data analyses

Analysis of the DGGE banding profile was performed with the software package BioNumerics v6.6 (Applied Maths, Belgium). Band standardization was carried out automatically by the program, but was always confirmed visually with changes made when necessary. Subsequently, the program constructed a matrix that incorporated the position and intensity of each band. Briefly, the matrix containing both band position and intensity were processed in a spreadsheet and transformed into relative abundance. The relative abundance matrix was uploaded to R, log_10_ (*x*+1) transformed and a distance matrix constructed with the Bray Curtis similarity coefficient using the vegdist() function in the vegan package [25] in R (version 2.11.1; http://www.r-project.org/). Variation in composition was visualised using principal coordinates analysis (PCO) with the cmdscale() function in R. Differences in the bacterial composition of RAS and water supply were tested using the adonis() function in vegan.

Pyrosequencing libraries were first analysed using QIIME (Quantitative Insights Into Microbial Ecology) (http://qiime.sourceforge.net/). First, data was filtered using the split_libraries.py script, which removed forward primers, barcodes and reverse primers. Sequences shorter than 200 base pairs were also removed. Operational taxonomic units (OTUs) were selected (97% similarity) using the pick_otus.py script with the 'usearch_ref' method and the most recent Greengenes release (Greengenes 12_10; http://qiime.wordpress.com/2012/10/16/greengenes-12_10-is-released/). Chimera were identified and removed using de novo and reference based chimera checking based on a reference fasta file from the Greengenes 12_10 release. Representative sequences were selected using the pick_rep_set.py script with the 'most_abundant' method and taxonomic identity was determined using the assign_taxonomy.py script with the Ribosomal Database Project (RDP) method [26]. We used the make_out_table.py script in QIIME to produce an OTU by sample table containing the abundance and taxonomic assignment of all OTUs. Unique OTUs were identified by assigning them to arbitrary numbers. This table was uploaded to R and non-bacteria, chloroplasts and mitochondria were removed from the analysis. Rarefaction curves were made for each sampling compartment using a self-written function in R [14]. Variation in OTU composition was visualised using principal coordinates analysis following the same method used for DGGE band data. Variation in the relative abundance of the most abundant bacterial taxa (two phyla, eight classes and ten orders) was assessed using bar plot graphs. The relative abundance was calculated considering the total of reads for each taxonomic level. In addition to this, OTUs taxonomically classified into genera known to be fish pathogens [27-30] (Data S1) were selected and their phylogeny investigated. BLAST search (http://www.ncbi.nlm.nih.gov/) was used to obtain the closest relatives of selected OTUs (pathogens and abundant taxa, i.e., where the number of sequences > 400). These sequences were also aligned using ClustalW and a phylogenetic tree was constructed using the neighbour-joining method in Mega 5.1 (http://www.megasoftware.net/). The evolutionary distances were computed using the Maximum Composite Likelihood method with a gamma distribution (four categories) and 500 bootstraps. 

The DNA sequences generated in this study were submitted to the NCBI SRA: Accession number SRP026529.

## Results and Discussion

### Bacterial Community Profiles

**Figure 2:**
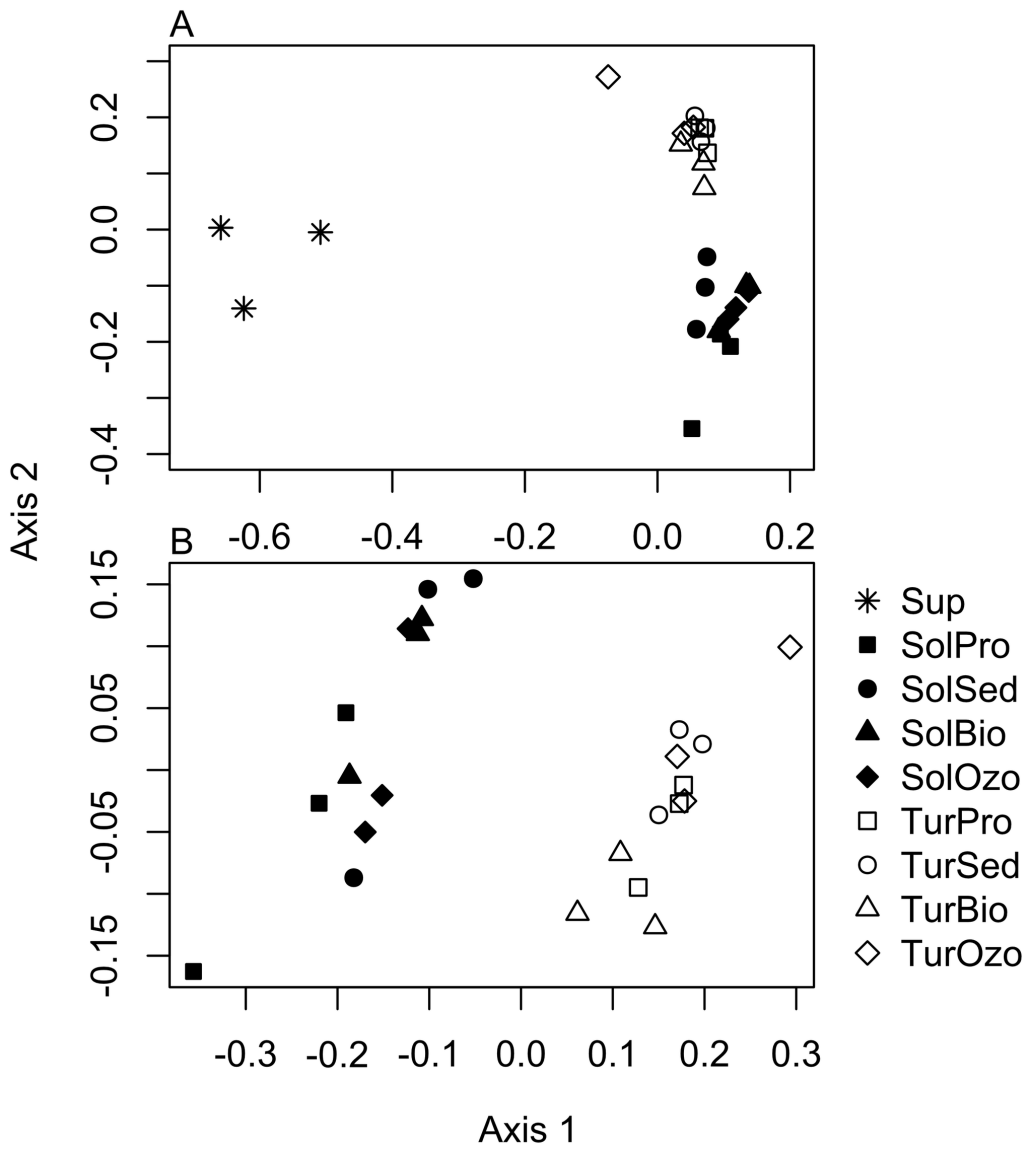
Ordination diagrams (PCO) of the bacterial community based on DGGE profile. A) in all sampling compartments (water supply (Sup), turbot production tank (TurPro), turbot sedimentation tank (TurSed), turbot biofilter tank (TurBio), turbot ozone tank (TurOzo), sole production tank (SolPro), sole sedimentation tank (SolSed), sole biofilter tank (SolBio), sole ozone tank (SolOzo)); B) only in turbot and sole sampling compartments (without water supply).

In this study, temperature, salinity and pH were similar in both RAS systems (Table 1). However, ammonia, nitrites and nitrates were present in lower concentrations in water samples collected from the RAS employed for sole production. The sole RAS also showed the highest values for solid particles and TOC. In line with these analyses, the water of different fish cultures also showed distinct microbial communities. The PCO ordination analysis of bacterial DGGE profiles (Figure 2) showed clear separation of three main groups in the ordination representing the water supply and the RAS compartments for turbot and sole production (Figure 2A). The differences in bacterial composition were highly significant (F_8,18_ = 6.94, P < 0.001, R^2^ = 0.755) among groups. The primary axis of variation of the PCO in Figure 2A revealed that bacterial communities from the water supplied to the RAS and the samples collected from the compartments of the RAS clearly differed. In order to gain a better insight on how the bacterial communities differed between the two fish RAS and among the different compartments of each RAS, a new PCO was performed (Figure 2B) excluding the samples from water supply. The second PCO (Figure 2B) confirmed the differences between the two fish RAS, as illustrated by the primary axis of variation, with a significant difference in composition among groups being recorded (F_7,16_ = 7.48, P < 0.001, R^2^ = 0.766). Previous studies have shown that gut fish microbes may colonize the biofilm of marine aquaculture systems and that their microbial composition will depend on the fish species being cultured [6,31]. In fact, fish faeces and unconsumed feed are important parameters controlled in RAS. Large amount of organic materials form suspended particles, which support the growth of heterotrophic bacteria. This adversely affects nitrifiers and increases concentrations of ammonia, nitrites and nitrates and may trigger the growth of pathogenic microorganisms [32-34]. However, in contrast to current knowledge [35,36], neither solid particulates nor TOC were associated with increased values of dissolved nitrogen in turbot RAS (Data S2). Sole RAS compartments showed the highest concentration of solid particulates and the lowest levels of dissolved nitrogen (Table 1). The PCO did not show pronounced separation between bacterial communities from different RAS compartments with the exception of the sedimentation compartment of the turbot RAS (Data S3).

**Table 1:** Mean values and standard deviation of temperature, pH, dissolved oxygen (DO), salinity, ammonia, nitrites, nitrates, bromine residuals, suspended particles, sulfates, phosphates and total organic carbon (TOC) in the aquaculture system [water supply (Sup), turbot production tank (TurPro), turbot sedimentation tank (TurSed), turbot biofilter tank (TurBio), turbot ozone tank (TurOzo), sole production tank (SolPro), sole sedimentation tank (SolSed), sole biofilter tank (SolBio), sole ozone tank (SolOzo)].

	**Temperature**	**pH**	**DO**	**Salinity**	**Ammonia**	**Nitrites**	**Nitrates**	**Bromine residuals**	**S. Particules**	**Sulfates**	**Phosphates**	**TOC**
	**(C°)**		**(mg /L)**		**(mg /L)**	**(mg /L)**	**(mg /L)**	**(mg /L)**	**(mg /L)**	**(mg /L)**	**(mg /L)**	**(mg /L)**
**Sup**	17.60 ± 0.08	7.40 ± 0.02	6.87 ± 0.09	24 ± 0	1.47 ± 0.05	0.80 ± 0.14	<10	0.06 ± 0.01	4.80 ± 1.23	1782.00 ± 13.64	<10	2.10 ± 0.26
**SolPro**	18.40 ± 0.00	6.77 ± 0.08	13.37 ± 2.30	24 ± 0	1.03 ± 0.12	0.48 ± 0.06	32.67 ± 9.29	0.16 ± 0.01	7.60 ± 1.78	1766.33 ± 9.24	<10	8.00 ± 0.00
**SolSed**	18.60 ± 0.10	6.60 ± 0.07	10.93 ± 0.06	24 ± 0	1.40 ± 0.17	0.53 ± 0.04	36.00 ± 1.73	0.06 ± 0.01	185.27 ± 33.69	1787.33 ± 4.04	<10	6.67 ± 1.53
**SolBio**	18.43 ± 0.06	6.97 ± 0.01	6.73 ± 0.51	24 ± 0	0.73 ± 0.06	0.30 ± 0.00	36.00 ± 2.00	0.07 ± 0.01	20.80 ± 2.31	1745.67 ± 50.08	<10	9.67 ± 1.53
**SolOzo**	18.50 ± 0.00	7.18 ± 0.19	8.90 ± 0.00	24 ± 0	0.60 ± 0.17	0.27 ± 0.06	26.00 ± 10.82	0.13 ± 0.03	10.00 ± 3.94	1777.67 ± 12.66	<10	6.00 ± 0.00
**TurPro**	18.77 ± 0.06	6.76 ± 0.04	16.60 ± 2.43	24 ± 0	2.33 ± 0.12	1.67 ± 0.72	82.33 ± 2.08	0.06 ± 0.01	6.73 ± 1.36	1760.00 ± 39.89	<10	7.33 ± 0.58
**TurSed**	18.73 ± 0.06	6.69 ± 0.01	11.33 ± 0.15	24 ± 0	3.43 ± 0.49	1.23 ± 0.35	85.00 ± 15.62	0.13 ± 0.02	6.60 ± 1.60	1708.00 ± 129.32	<10	7.00 ± 1.00
**TurBio**	19.00 ± 0.00	6.88 ± 0.04	7.33 ± 0.06	24 ± 0	1.60 ± 0.17)	1.57 ± 0.15	86.00 ± 10.54	0.11 ± 0.02	10.20 ± 2.95	1720.33 ± 22.37	<10	8.00 ± 1.00
**TurOzo**	18.80 ± 0.00	6.98 ± 0.01	8.73 ± 0.06	24 ± 0	1.73 ± 0.21	2.00 ± 0.65	57.33 ± 11.72	0.15 ± 0.06	5.33 ± 2.14	1567.00 ± 208.32	<10	7.67 ± 0.58

The sign < indicates values below detection limit.

### Overall Assessment of Bacterial Composition in RAS

**Figure 3:**
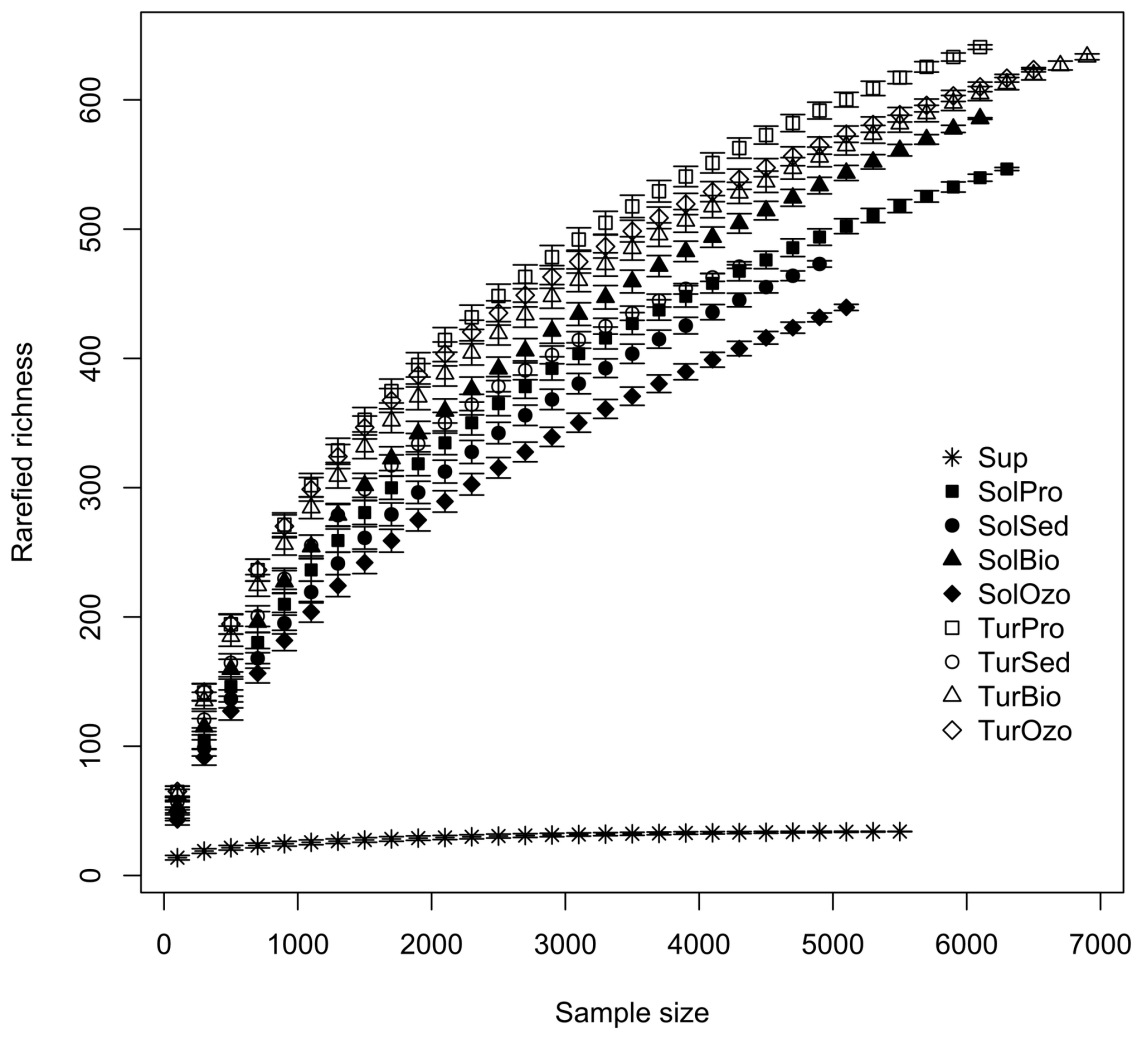
Bacterial richness. Rarefied OTU richness in all sampling compartments (water supply (Sup), turbot production tank (TurPro), turbot sedimentation tank (TurSed), turbot biofilter tank (TurBio), turbot ozone tank (TurOzo), sole production tank (SolPro), sole sedimentation tank (SolSed), sole biofilter tank (SolBio), sole ozone tank (SolOzo)).

Barcoded pyrosequencing analysis yielded 5553, 24214 and 22786 sequence reads for water supply, turbot and sole RAS compartments, respectively. In terms of bacterial community diversity, the water in the turbot RAS showed the highest bacterial richness (Figure 3). Controlling for sample size (*n* = 4300 individual sequences), OTU richness varied from 562.58 ± 7.58 OTUs in the shallow raceway tank to 527.65 ± 8.40 OTUs in the biofilter tank of turbot RAS. OTU richness of the sole RAS varied from 504.45 ± 7.70 OTUs in the biofilter tank to 445.03 ± 5.34 OTUs in the sedimentation tank. The water supply used in the RAS had the lowest richness, 33.24 ± 0.82 OTUs. Although the ozone compartment in sole RAS exhibited relatively low richness, the ozone compartment in turbot had similar values to those obtained in other compartments.

**Figure 4:**
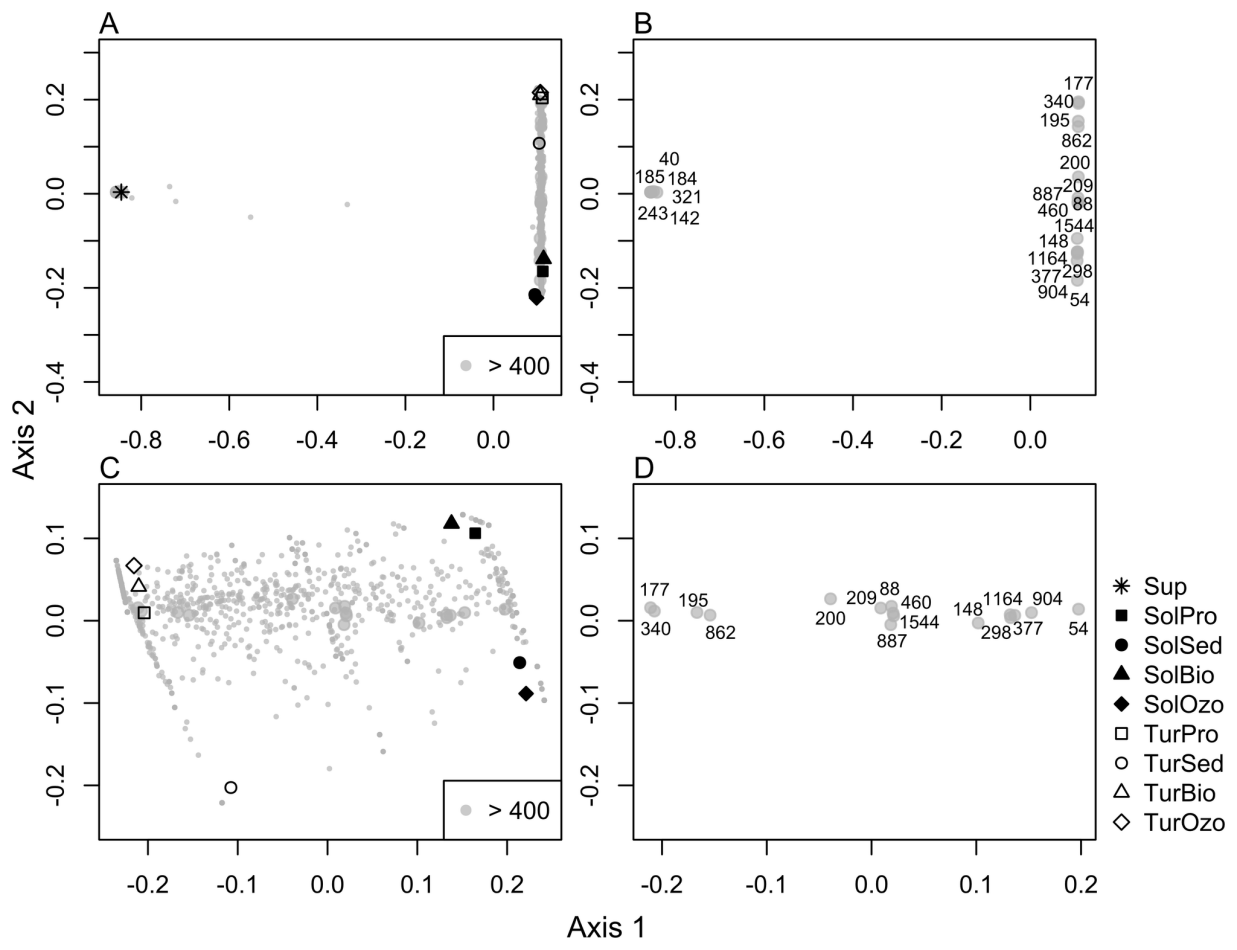
Ordination diagrams (PCO) of the bacterial community based on barcoded pyrosequencing data. (A) in all sampling compartments; (B) the most abundant OTU’s associated to all sampling points; (C) only in turbot and the sole sampling compartments (without water supply); (D) the most abundant OTUs associated to turbot and the sole sampling compartments.

In line with the PCO of DGGE profiles (Figure 2), the ordination of barcoded-pyrosequencing data (OTU composition) showed that the water supply had the most distinct microbial community (Figure 4A). The PCO comprising only turbot and sole production systems also showed clear differences between the two RAS (Figure 4C) with a range of abundant OTUs (> 400 sequences) specific to each system (Figure 4C and D). Only few bacterial OTUs (~4) were found in the water supply and RAS. These results indicate that bacterial populations entering the system through the water supply are probably out-competed by those already established in turbot and sole RAS.

**Figure 5:**
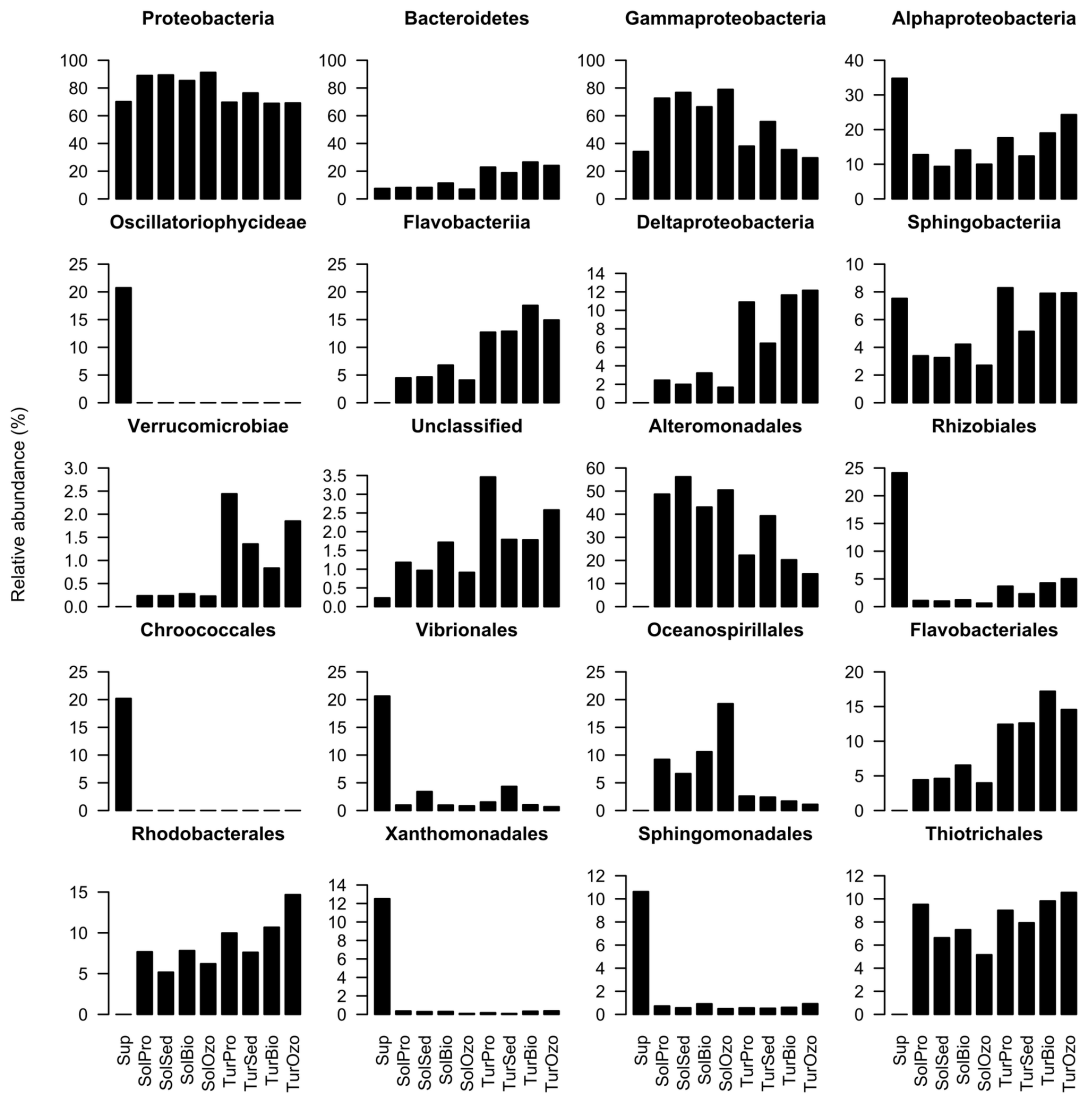
Dominant bacterial groups. Relative abundance of the most dominant bacterial groups (2 phyla, 8 classes and 10 orders) in each sampling compartments (water supply (Sup), turbot production tank (TurPro), turbot sedimentation tank (TurSed), turbot biofilter tank (TurBio), turbot ozone tank (TurOzo), sole production tank (SolPro), sole sedimentation tank (SolSed), sole biofilter tank (SolBio), sole ozone tank (SolOzo)). Groups are present from the most abundant to the least abundant.

The overall taxonomic analyses grouped bacterial sequences into twenty-four phyla, forty-two classes and sixty-one orders. At the phylum level, about 8% of OTUs remained unclassified. Figure 5 shows the relative abundance of the most dominant bacterial groups (≥ 400 reads). In agreement with the PCO analysis, the water supply had the most distinct composition, showing the highest abundance values for *Oscillatoriophycideae* (20.7%), *Rhizobiales* (24.1%), *Chroococcales* (20.2%), *Vibrionales* (20.6%), *Xanthomonadales* (12.5%) and *Sphingomonadales* (10.6%) (Figure 5). *Proteobacteria* was the most abundant phylum in all fish sampling compartments and displayed a slight dominance in sole RAS. In general, its relative abundance ranged between 70% and 90%. This phylum is widely dispersed in marine environments and plays an important role in the processes of nutrient cycling and mineralization of organic compounds [37,38]. *Bacteroidetes* was the second most abundant phylum with a relative abundance ranging from 7% to 11% in sole RAS and 18% to 26% in turbot RAS compartments. The *Bacteroidetes* (previously *Cytophaga-Flexibacter-Bacteroides*) are dominant members of marine heterotrophic bacterioplankton and are frequently found colonizing macroscopic organic matter particles (marine snow) [39]. Further differences were also observed at lower taxonomic levels between turbot and sole RAS compartments (e.g., *Gammaproteobacteria*, *Alphaproteobacteria*, *Deltaproteobacteria*, *Oceanospirillales* and *Verrucomicrobiae*) (Figure 5). 

### Composition Analysis of Dominant OTUs

The dominance analysis revealed six OTUs found in the water supply: 40 (unknown *Chroococcales*), 142 (unknown *Sphingobacteriales*), 184 (*Sphingomonas*), 185 (*Stenotrophomonas*), 243 (*Aliivibrio*) and 321 (*Phyllobacterium*) (Figure 4 and Table 2). However, none of these OTUs were dominant in the RAS compartments.

**Table 2:** Taxonomic affiliation of the most abundant OTUs (>400 reads) in water supply, turbot and sole RAS-SRS, and their closest relatives (using Blast) with the respective accession number, sequence identity (Sq ident) and source.

**OTU**		**Reads**		**Class**	**Order**	**Family**	**Genus**	**Acession**	**Sq ident**	**Source**
	**Tur**	**Sol**	**Sup**							
40	0	0	1079	*Oscillatoriophycideae*	*Chroococcales*	*Xenococcaceae*	Unclassified	EU780251	99	disease affected *Turbinaria mesenterina* colony
54	10	681	0	*Gammaproteobacteria*	*Oceanospirillales*	*Oleiphilaceae*	Unclassified	JN092240	100	gut *Nephrops norvegicus*
88	281	361	0	*Flavobacteriia*	*Flavobacteriales*	*Flavobacteriaceae*	*Olleya*	JN175350	100	seawater particulates, water temperature 5°C
142	0	0	418	*Sphingobacteriia*	*Sphingobacteriales*	Unclassified	Unclassified	FJ178015	95	*Austrocochlea concamerata*
148	196	559	0	*Gammaproteobacteria*	*Alteromonadales*	*Colwelliaceae*	Unclassified	KC756863	100	*Paralichthys olivaceus*
177	466	9	0	*Alphaproteobacteria*	*Rhizobiales*	*Hyphomicrobiaceae*	Unclassified	FR647917	99	seawater
184	0	1	564	*Alphaproteobacteria*	*Sphingomonadales*	*Sphingomonadaceae*	*Sphingomonas*	JQ229600	100	Crater Cirque Lake
185	0	0	679	*Gammaproteobacteria*	*Xanthomonadales*	*Xanthomonadaceae*	*Stenotrophomonas*	EU438980	100	paper mill pulps containing recycled fibres
195	756	97	0	*Flavobacteriia*	*Flavobacteriales*	*Flavobacteriaceae*	*Kordia*	FJ015036	100	turbot larval rearing unit, tank surface
200	1405	1060	0	*Gammaproteobacteria*	*Thiotrichales*	*Thiotrichaceae*	*Leucothrix*	GU451651	100	macroalgal surface
209	347	398	0	*Alphaproteobacteria*	*Rhodobacterales*	*Rhodobacteraceae*	*Phaeobacter*	NR_043888	100	*Phaeobacter arcticus* DSM 23566
243	7	8	998	*Gammaproteobacteria*	*Vibrionales*	*Vibrionaceae*	*Aliivibrio*	AY292946	100	*Aliivibrio fischeri*
298	314	1481	0	*Gammaproteobacteria*	*Oceanospirillales*	*Oleiphilaceae*	Unclassified	EF215752	99	inert artificial surfaces submerged in marine water
321	7	0	1003	*Alphaproteobacteria*	*Rhizobiales*	*Phyllobacteriaceae*	*Phyllobacterium*	JQ316262	100	soil from Fazenda Nova Vida
340	1749	28	0	*Deltaproteobacteria*	PB19	Unclassified	Unclassified	EU283402	95	*Phaeobacter arcticus* DSM 23566
377	1060	4681	0	*Gammaproteobacteria*	*Alteromonadales*	*Pseudoalteromonadaceae*	*Pseudoalteromonas*	HQ401050	100	biofilm from surface of coral reef
460	1526	1926	0	*Gammaproteobacteria*	*Alteromonadales*	*Colwelliaceae*	*Thalassomonas*	HM237288	98	*Thalassomonas* sp. M-M1
862	528	87	0	*Flavobacteriia*	*Flavobacteriales*	*Flavobacteriaceae*	*Polaribacter*	EU586892	100	aquaculture seawater
887	362	421	0	*Gammaproteobacteria*	*Alteromonadales*	*Colwelliaceae*	*Thalassomonas*	HM237288	100	*Thalassomonas* sp. M-M1
904	64	418	0	*Gammaproteobacteria*	*Alteromonadales*	*Pseudoalteromonadaceae*	*Pseudoalteromonas*	FJ154991	99	ocean water
1164	143	687	0	*Gammaproteobacteria*	*Alteromonadales*	*Pseudoalteromonadaceae*	*Pseudoalteromonas*	AB257337	100	*Pseudoalteromonas mariniglutinosa*
1544	297	378	0	*Gammaproteobacteria*	*Alteromonadales*	*Colwelliaceae*	*Thalassomonas*	HM237288	98	*Thalassomonas* sp. M-M1

Reads indicates the number of sequences obtained for each OTU in water supply (Sup), turbot (Tur) and sole (Sol).

**Figure 6:**
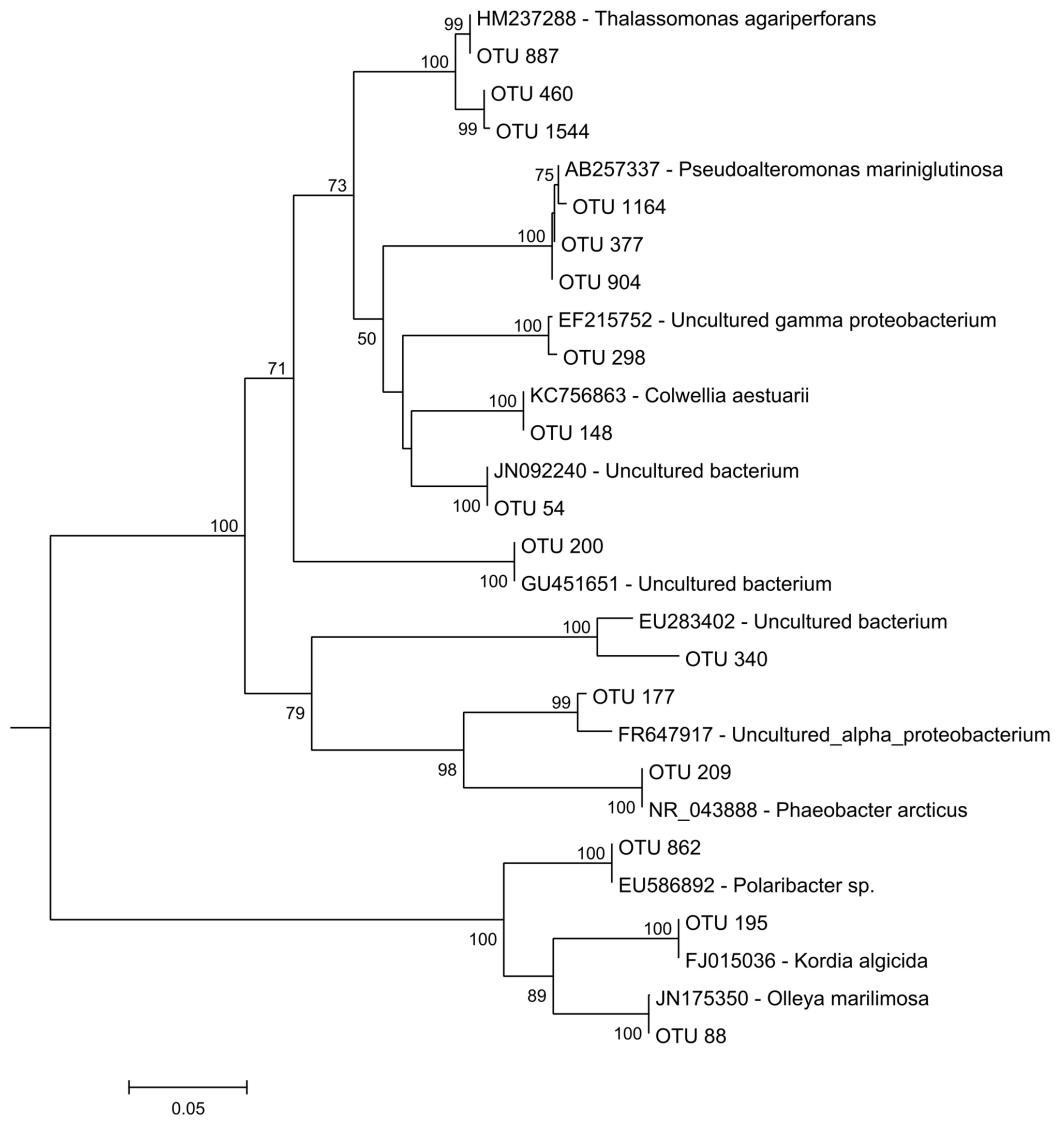
Neighbour-joining phylogenetic tree (16S rRNA gene sequences) of the most dominant OTUs in turbot and sole RAS and their closest relatives (accession number-species). Bootstrap values generated from 500 replicates. Values lower than 50% were omitted.

The most abundant OTUs in turbot RAS compartments were assigned to *Kordia* (OTU 195), *Polaribacter* (OTU 862), unknown *Hyphomicrobiaceae* (OTU 177) and unknown *PB19* (OTU 340) group (Figure 4 and Table 2). Previous studies on *Kordia* spp. have shown that members of this genus can exhibit algicidal activity and produce extracellular proteases responsible for the cell lysis of diatom species [40]. *Kordia* spp. was also previously detected in biofilter media employed in RAS for the culture of goldfish *Carassius auratus* [41]. Goméz-Consarnau et al. [42] showed that some *Polaribacter* species have the ability to produce specific bacterial compounds (namely rhodopsins) that induce growth when associated with light (photoheterotrophy). OTU 177 was classified as an unknown member of the family *Hyphomicrobiaceae* and was closely related with a partial sequence of a 16S rRNA gene retrieved from marine bacterioplankton communities after environmental disturbance [43] (Figure 6). Species in this family have been recognized as important methylotrophic denitrifiers in fluidized bed reactors and activated sludge [44,45]. The most dominant OTU in the turbot RAS was OTU 340 (1749 reads). This OTU was classified as unknown PB19, and according to Blast (http://www.ncbi.nlm.nih.gov/) was related to an uncultured bacterium [GenBank accession number (acc.) EU283402] isolated from activated sludge produced by an aerated submerged membrane bioreactor for domestic wastewater treatment [46] (Figure 6).

OTUs 54, 377, 904 and 1164 were the most abundant in the sole RAS compartments, and were classified as *Pseudoalteromonas* spp. (*Alteromonadales*), except for OTU 54 that was identified as an unclassified *Oceanospirillales* (Figure 4 and Table 2). According to Wawrik et al. [47] in a study of assimilatory nitrate utilization by bacteria on the West Florida Shelf, *Alteromonadales* and *Oceanospirillales* orders were identified as relevant marine heterotrophic bacteria involved in the uptake of dissolved inorganic nitrogen (DIN). Therefore, their higher abundance in sole RAS compartments may be in part responsible for the lower DIN values observed in this system when compared to the turbot RAS (Table 1). Members of the order *Oceanospirillales* are also often involved in symbiotic interactions with marine animals; however, their putative functional role in fish is yet to be determined [48]. In line with the higher-taxon analysis (Figure 5), the most abundant OTUs (OTUs 1164, 377 and 904) detected in sole RAS were related to *Pseudoalteromonas mariniglutinosa* (acc. AB257337) (Figure 6). A member of the *Alteromonadales* order previously isolated from an organically enriched sediment below fish farms [49]. Recently, Aranda et al. [50] showed that *Pseudoalteromonas* sp. strains (related to *P. mariniglutinosa*) could be used as *Vibrio*-biocontrol agents, as they were able to produce a putatively novel class of bacteriostatic compounds. 

The dominant OTUs 88 (*Olleya*), 148 (unclassified *Alteromonadales*), 200 (*Leucothrix*), 209 (*Phaeobacter*), 298 (unclassified *Oceanospirillales*), 460 (*Thalassomonas*), 887 (*Thalassomonas*) and 1544 (*Thalassomonas*) were abundant in both RAS (Figure 4D). OTU 88 was assigned to the genus *Olleya* and was closely related to *Olleya marilimosa* (Figure 6) isolated from a Danish turbot farm [51]. This bacterium produces an exopolysaccharide in liquid media which may contribute to the capture, sinking and mineralization of organic substances in natural marine environments [38,52]. OTUs 148 and 298 were closely related with *Colwellia aestuarii* (acc. KC756863) and an unknown member of the *Oceanospirillales* order (acc. EF215752), respectively (Figure 6). These taxa comprise marine bacterial guilds often associated with nitrate reduction [47,53]. OTU 200 was classified as *Leucothrix* and was closely related to an epibiont bacteria (acc. GU451651). The epibiont *Leucothrix mucor* is the most studied member of this genus and is known to cause fouling diseases in prawns, although it is not considered to be a true pathogen [54].

Interestingly, our analysis detected OTUs phylogenetically related to taxa comprising bacterial species with potential activity against several aquaculture pathogens. OTUs 460, 887 and 1544 were closely related with *Thalassomonas agariperforans* previously isolated from marine sand [55]. Recently, Torres et al [56] showed that a member of this genus (*Thalassomonas* sp. PP2-459) isolated from a bivalve hatchery was capable of degrading N-acylhomoserine lactone (AHL) signal molecules (quorum quenching). The degradation of this molecule may affect the quorum sensing system of potential pathogens. Quorum sensing is a mechanism that allows bacteria to coordinate the expression of certain genes (including virulence genes) in response to the presence of small signal molecules such as AHL [57]. Recent studies showed that the use of bacterial strains with quorum quenching activity might be a useful strategy to biocontrol aquaculture pathogens [58,59]. We also detected an abundant OTU (209) closely related to *Phaeobacter arcticus*. Recent studies showed that members of the genus *Phaeobacter* can also have antagonistic activity against known aquaculture pathogens [60-62]. 

### Phylogenetic analyses of potential fish pathogens

**Figure 7:**
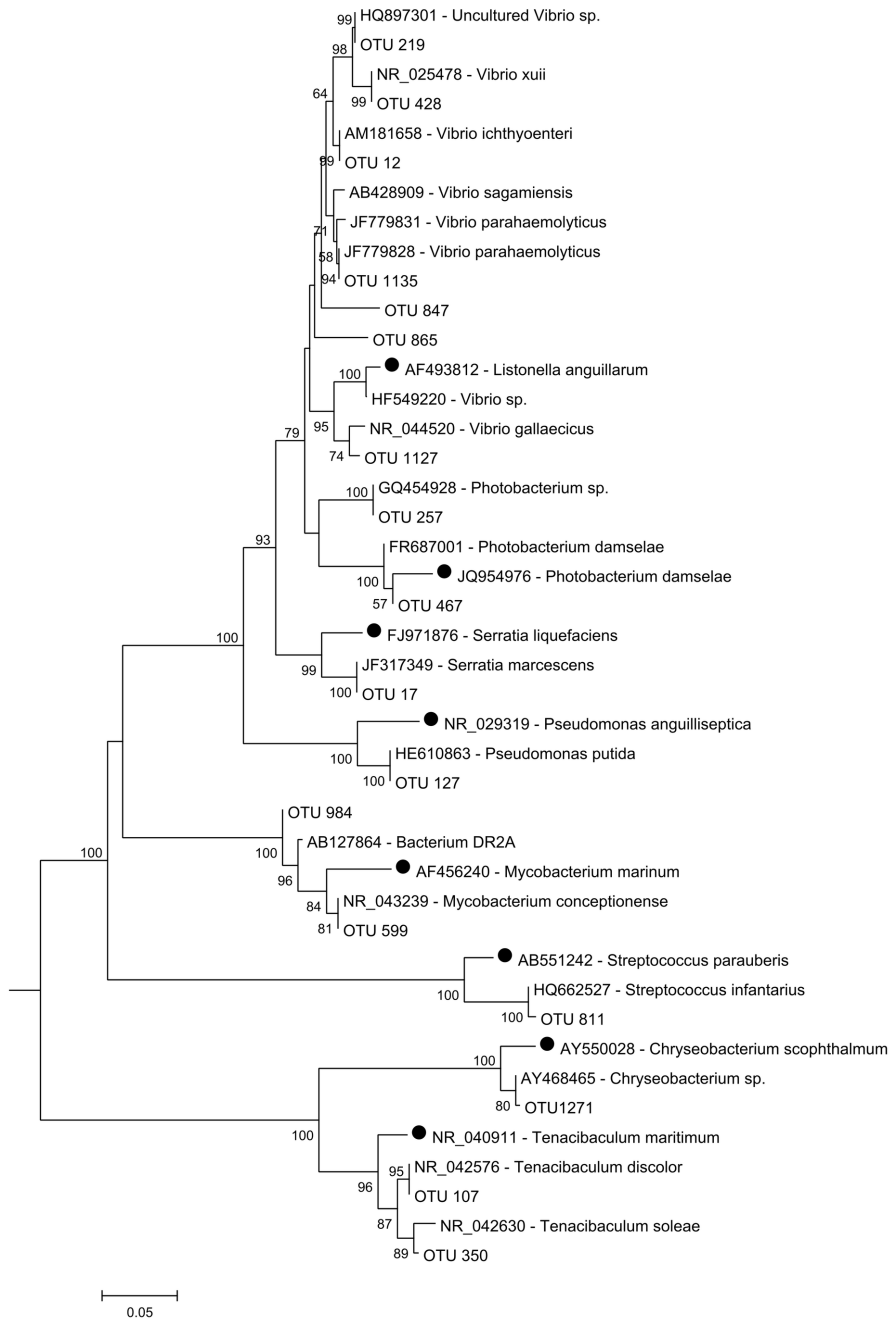
Neighbour-joining phylogenetic tree (16S rRNA gene sequences) of OTUs related potential fish pathogens and their closest relatives (with accession number). Black circles indicate the most frequent pathogens described in literature. Bootstrap values generated from 500 replicates. Values lower than 50% were omitted.

In order to understand the distribution of potential fish pathogens in the aquaculture system studied, a list of the most frequent bacterial pathogens responsible for fish diseases in aquacultures located in Europe was compiled [27-30] (Data S1). The 16S rRNA gene sequences of these bacteria were then phylogenetically compared with related OTUs (same genera) detected in this study (potential fish pathogens) and their closest GenBank relatives (blastn tool - http://blast.ncbi.nlm.nih.gov/) (Figure 7). In addition to this, the spatial distribution and the relative abundance of potential pathogens in RAS (turbot and sole) were investigated (Table 3). OTUs 257 and 467 were closely related to an unknown *Photobacterium* sp. and *P. damselae*, respectively. *P. damselae* subsp. *piscicida* is one of the most common pathogens associated with sole aquaculture [63,64]. However, in our study, *P. damselae* (OTU 467) was only detected in the turbot RAS compartments, and was more abundant in production tanks and the sedimentation filter. In contrast, we detected OTUs closely related to *T. discolor* (OTU 107) and *T. soleae* (OUT 350) in nearly all turbot and sole RAS compartments. *T. soleae* and *T. discolor* are known fish pathogens responsible for tenacibaculosis disease and were first isolated from diseased sole (*S. senegalensis* and *S. solea*) and turbot (*S. maximus*) in Spain [65,66]. Eight OTUs were classified (OTUs 12, 219, 428, 503, 847, 865, 1127 and 1135) as members of the genus *Vibrio* (data not shown). OTUs 12, 1135, 1127 and 428 were closely related with 16S rRNA gene sequences of *V. ichthyoenteri, V. parahaemolyticus*, *V. gallaecicus* and *V. xuii*, respectively (Figure 7). All these *Vibrio* species were isolated from marine aquaculture environments. However, only *V. ichthyoenteri* and *V. parahaemolyticus* are potentially pathogenic, as they are often associated with diseased animals [67,68]. These species were detected in nearly all RAS compartments. Our analysis also detected an OTU with strong homology to *S. marcescens* (OTU 17). This species is known to be an opportunistic pathogen previously detected in brackish and freshwaters and is a causative disease agent in natural population of white perch [69]. This potential pathogen was also isolated from diseased tilapia fish (*Oreochromis niloticus*) in Malaysia [70].

**Table 3:** Values of relative abundance (%) of potential fish pathogens detected in water supply (Sup), turbot production tank (TurPro), turbot sedimentation tank (TurSed), turbot biofilter tank (TurBio), turbot ozone tank (TurOzo), sole production tank (SolPro), sole sedimentation tank (SolSed), sole biofilter tank (SolBio), sole ozone tank (SolOzo).

**Potential fish pathogen**	**OTU**	**Sup**	**SolPro**	**SolSed**	**SolBio**	**SolOzo**	**TurPro**	**TurSed**	**TurBio**	**TurOzo**
***Chryseobacterium scophthalmum***	1271	ND	ND	ND	0.016	ND	ND	ND	ND	ND
***Mycobacterium conceptionense***	599	ND	ND	ND	0.016	ND	ND	0.023	ND	0.015
***Photobacterium damselae***	467	ND	ND	ND	ND	ND	0.064	0.069	0.014	0.030
***Pseudomonas putida***	127	0.018	0.079	0.039	0.065	0.114	0.081	0.092	0.141	0.227
***Serratia marcescens***	17	0.990	ND	0.039	ND	ND	ND	0.046	0.042	0.030
***Streptococcus infantarius***	811	ND	ND	ND	ND	ND	ND	0.023	ND	ND
***Tenacibaculum discolor***	107	ND	0.189	0.138	0.114	0.152	0.307	0.253	0.212	0.242
***Tenacibaculum soleae***	350	ND	0.031	0.039	0.033	ND	0.081	0.069	0.014	0.015
***Vibrio ichthyoenteri***	12	ND	0.252	0.454	0.147	0.133	0.275	1.034	0.198	0.167
***Vibrio parahaemolyticus***	1135	ND	0.031	0.020	0.016	0.038	0.032	0.184	0.014	ND

ND – Not detected

Some selected OTUs in this study were closely related to potential new fish pathogens. For example, OTU 127 was close related to *P. putida*. This species is known to be an opportunistic human pathogen, although a previous report suggests that *P. putida* may also be a disease causative agent in rainbow trout aquaculture [71]. OTUs 599 and 881 were closely related to *Mycobacterium conceptionense* and *Streptococcus infantarius*, respectively. These species can be found colonizing human and environmental samples, but there is no previous report on their occurrence in aquaculture systems. However, it is important to note that the genera *Mycobacterium* and *Streptococcus* comprise several members able to cause mycobacteriosis and streptococcosis diseases among both wild and captive fishes worldwide [72,73]. 

Despite the presence of pathogens, no diseased fish were registered during the study period. The composition and relative abundance analysis of the potential bacterial pathogens in turbot and sole RAS (Table 3) indicated that OTUs closely related to fish pathogens were present at a low abundance (low infective concentration) in both RAS. Fish density and infectious dose have been considered as key factors to control fish mortality [74,75]. Possibly, the high abundance of naturally occurring antagonistic strains detected in this study (e.g., bacterial populations closely related to known antagonistic strains belonging to the genus *Thalassomonas* and *Phaeobacter*; see above) may have contributed to suppress the development of potential fish pathogens. 

### Critical evaluation of barcoded pyrosequencing of 16S rRNA gene fragments for fish disease detection

The results obtained in this study were critically evaluated to underline the advantages and disadvantages of using barcoded pyrosequencing of 16S rRNA gene fragments for monitoring potential fish pathogens in aquaculture systems. The 16S rRNA gene is widely used in phylogenetic studies and is an important marker for molecular diagnostics and molecular ecology studies. However, this gene may fail to provide a sufficient phylogenetic resolution or a correct classification of some bacterial pathogens. For example, the 16S rRNA gene fragments used in this study, are unable to resolve differences between two different *P. damselae* subspecies, namely, *damselae* and *piscicida* [76]. These subspecies may, however, cause very different infections in a variety of fish species [28,63]. In addition to this, *P. damselae* subsp. *piscicida* is more infectious. This bacterium is the causative agent of pasteurellosis, one of the most common diseases in marine fish farms in the Iberian Peninsula [64,77]. The same problem may be observed at the species level for other bacteria. The 16S rRNA gene phylogeny provides an accurate classification of *Vibrio* species at family and genus level, but due to the high similarity between distinct species, it fails to provide an accurate identification at species level [78]. This was the case for *V. ichthyoenteri* (OTU 12)*, V. parahaemolyticus* (OTU 847 and 1135), *V. gallaecicus* (OTU 1127) and *V. xuii* (OTU 428) detected in this study. Thompson et al. [79], showed that *V. ichthyoenteri* and *V. scophthalmi* and *V. nereis* and *V. xuii* had 99% 16S rDNA sequence similarity but only shared 90% *recA* gene sequence similarity. According to Osorio and Klose [76], *V. parahaemolyticus* and *V. alginolyticus* sequences were 99.8% similar using the 16S rRNA marker gene while using partial *toxR* gene showed only 61.7% similarity. Beaz-Hidalgo et al. [80] also showed that the *recA* gene is a better genetic marker to discriminate *V. gallaecicus* than the 16S rRNA gene. Several other studies reported that the degree of resolution obtained with the 16S rRNA gene is not sufficiently robust for phylogenetic analysis of some known bacterial fish pathogens [81-86].

Another additional problem is that none of the next generation sequencing technologies developed until now can provide long sequence reads of gene fragments (pyrosequencing provides the longest fragment size ≤ 600 bp). Very often long stretch nucleotide sequences or complete nucleotide sequences of the 16S rRNA gene (~1,500 bp) are necessary for clear differentiation of closely related species. Other phylogenetic markers can be used, however, while the 16S rRNA gene sequence database (The Ribosomal Database Project) has currently over 2,765,278 sequences, other genetic databases are substantially smaller and incomplete (lacking representative sequences for all taxa and limited numbers of sampled ecotypes) (http://rdp.cme.msu.edu/; Accessed 30 July 2013). Therefore, this problem will hamper a fast identification of a range of bacterial species or the establishment of relationships between the sequence retrieved and their closest relative in the GenBank database. Currently, this approach allows us to associate 16S rRNA gene sequences with other sequences in the database previously detected in a specific environment or case of study (e.g., ecotypes and case studies about emergent causative agents of disease outbreaks).

## Conclusion

In this study we applied a combined DGGE and pyrosequencing approach to assess the structural variation and composition of bacterial communities in RAS for turbot and sole production. The DGGE approach revealed significant structural differences between bacterial communities from turbot and sole RAS. This result suggests a strong fish species-specific effect on bacterial communities of both RAS studied. The pyrosequencing approach provided fundamental information about the bacterial composition and pathogen load in different RAS compartments. However, despite detecting potential fish pathogens in sole and turbot RAS, no symptomatic fish were observed during this study. Our goal in the future should be to identify the triggering mechanism(s) causing fish infection and disease progress in aquaculture facilities. The use of a high throughput sequencing approach using the 16S rRNA gene allowed us an unprecedented means to detect pathogens in the aquaculture systems studied. However, while the use of the 16S rRNA gene is commonly recognized to be a suitable option when studying microbial composition, it may not be optimal for detecting some bacterial groups, including potential fish pathogens. Therefore, data obtained needs to be carefully examined and critically evaluated in terms of the level of resolution provided by the 16S RNA gene. Other conventional molecular tools may be used in combination with this technology to ensure the correct identification of some specific bacteria [e.g. Real Time PCR, Fluorescence In Situ Hybridization (FISH) and Isothermal DNA amplification], as well as the use of a different phylogenetic marker.

High-throughput sequencing technologies are now widely used in scientific research and, given the rapid reduction in their operating costs, it is likely that they will soon be routinely used to screen for pathogens and compare bacterial communities in aquaculture systems.

## Supporting Information

Data S1 **Most frequent bacterial pathogens in aquaculture systems of turbot and sole production described in literature and the pathogen species identified in this study.**
(DOCX)

Data S2 **Principal Coordinates Analysis of the RAS bacterial communities.** Water parameters (ammonia, nitrites, nitrates, bromine residuals, sulfates, total organic carbon, temperature, pH, dissolved oxygen, suspended particles and salinity) are represented by vectors.
(DOCX)

Data S3 **ANOSIM, pairwise test comparing among sampling compartments (global R=0.62).**
(DOCX)
