# Supplementary material for: Molecular Analysis of Bacterial Communities and Detection of Potential Pathogens in a Recirculating Aquaculture System for Scophthalmus maximus and Solea senegalensis
Source: PLoS One. 2013 Nov 21;8(11):e80847. doi: 10.1371/journal.pone.0080847 (PMC3836758; doi:10.1371/journal.pone.0080847)
Supplement: Data S1 — Most frequent bacterial pathogens in aquaculture systems of turbot and sole production described in literature and the pathogen species identified in this study. (DOCX) [file pone.0080847.s001.docx]

**Data S1.**

|  | **Common fish pathogens** | | **Putative fish pathogens** | |
| --- | --- | --- | --- | --- |
| **Fish pathogen species** | **(literature)** | | **(this study)** | |
|  | **Turbot** | **Sole** | **Turbot** | **Sole** |
| *Aeromonas salmonicida* | X |  |  |  |
| *Chryseobacterium scophthalmum* | X |  |  |  |
| *Mycobacterium conceptionense* |  |  | X | X |
| *Mycobacterium marinum* | X |  |  |  |
| *Photobacterium damselae* | X | X | X |  |
| *Pseudomonas anguilliseptica* | X | X |  |  |
| *Pseudomonas putida* |  |  | X | X |
| *Serratia liquefaciens* | X |  |  |  |
| *Serratia marcescens* |  |  | X | X |
| *Streptococcus infantarius* |  |  | X |  |
| *Streptococcus parauberis* | X |  |  |  |
| *Tenacibaculum discolor* |  |  | X | X |
| *Tenacibaculum maritimum* | X | X |  |  |
| *Tenacibaculum soleae* |  |  | X | X |
| *Vibrio anguillarum* | X | X | X | X |
| *Vibrio gallaecicus* |  |  | X |  |
| *Vibrio ichthyoenteri* |  |  | X | X |
| *Vibrio parahaemolyticus* |  |  | X | X |
| *Vibrio sagamiensis* |  |  | X | X |
| *Vibrio xuii* |  |  | X | X |
