# Supplementary figures and images for: Molecular Analysis of Bacterial Communities and Detection of Potential Pathogens in a Recirculating Aquaculture System for Scophthalmus maximus and Solea senegalensis
Source: PLoS One. 2013 Nov 21;8(11):e80847. doi: 10.1371/journal.pone.0080847 (PMC3836758; doi:10.1371/journal.pone.0080847)

**Data S2.**


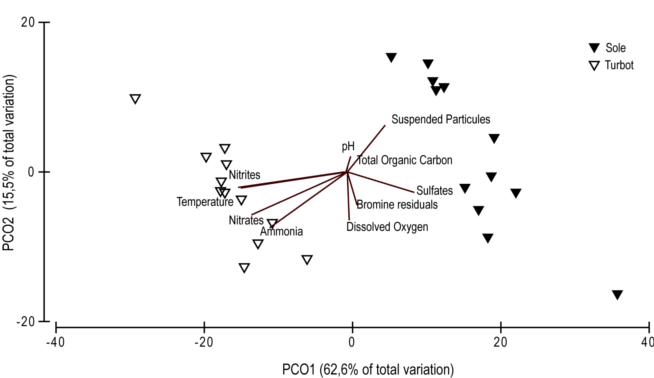

Supplement: Data S2 — Principal Coordinates Analysis of the RAS bacterial communities. Water parameters (ammonia, nitrites, nitrates, bromine residuals, sulfates, total organic carbon, temperature, pH, dissolved oxygen, suspended particles and salinity) are represented by vectors. (DOCX) [file pone.0080847.s002.docx]
