# Supplementary material for: Molecular Analysis of Bacterial Communities and Detection of Potential Pathogens in a Recirculating Aquaculture System for Scophthalmus maximus and Solea senegalensis
Source: PLoS One. 2013 Nov 21;8(11):e80847. doi: 10.1371/journal.pone.0080847 (PMC3836758; doi:10.1371/journal.pone.0080847)
Supplement: Data S3 — ANOSIM, pairwise test comparing among sampling compartments (global R=0.62). (DOCX) [file pone.0080847.s003.docx]

**Data S3**.

| **R statistic** | | | | | | | | | | |
| --- | --- | --- | --- | --- | --- | --- | --- | --- | --- | --- |
|  |  | **Sup** | **SolPro** | **SolSed** | **SolBio** | **SolOzo** | **TurPro** | **TurSed** | **TurBio** | **TurOzo** |
| **R statistic** | **Sup** | - |  |  |  |  |  |  |  |  |
|  | **SolPro** | 1 | - |  |  |  |  |  |  |  |
|  | **SolSed** | 1 | 0.222 | - |  |  |  |  |  |  |
|  | **SolBio** | 1 | 0.407 | 0.111 | - |  |  |  |  |  |
|  | **SolOzo** | 1 | 0.37 | 0.074 | 0.63 | - |  |  |  |  |
|  | **TurPro** | 1 | 1 | 1 | 1 | 1 | - |  |  |  |
|  | **TurSed** | 1 | 1 | 1 | 1 | 1 | 0.481 | - |  |  |
|  | **TurBio** | 1 | 1 | 1 | 1 | 1 | 0.148 | 0.593 | - |  |
|  | **TurOzo** | 1 | 1 | 1 | 0.96 | 1 | 0.481 | 0.556 | 0.444 | - |
